# When History Seems to Repeat Itself: Exposure to Perceived Lessons of the Past Influences Predictions About Current Political Events

**DOI:** 10.5334/pb.1075

**Published:** 2022-03-16

**Authors:** Djouaria Ghilani, Olivier Luminet, Olivier Klein

**Affiliations:** 1 Université libre de Bruxelles (ULB), BE; 2 Fonds National de la Recherche Scientifique (F.R.S.-F.N.R.S.), BE; 3 Université catholique de Louvain (UCLouvain), BE

**Keywords:** Historical analogies, predictions, politics, social cognition

## Abstract

The idea that the past holds lessons for the present, under the guise of *historical analogies*, is prevalent in political and public discourse. Those analogies are often accompanied by dire warnings befalling those who “forget” or otherwise neglect the powerful lessons of History—and would then be “doomed to repeat it”, as the saying goes. So, Would remembering history make it seem more OR LESS likely to repeat itself in the future? In other words, does exposure to specific lessons about past events, especially ones involving causal claims, affect how people expect real-life events to turn out? Four studies (three preregistered) tested this experimentally. In Studies 1 and 2, participants expected the same behavior (the US adopting a harsh stance against Iran in the Nuclear Treaty) to result in a more negative outcome when this current stance seemed to match a “lesson” they had read about the break-out of World War II (European leaders adopting a harsh approach against Germany in the 1919 Versailles Treaty vs. a conciliatory approach in the 1938 Munich Agreement). Studies 3 and 4 attempted to eliminate some confounds present in the first two studies and to generalize the effect to different events. While results varied across studies, an internal meta-analysis indicated that the analogical effect on predictions (d = –.08) tended to become stronger as participants’ knowledge about the target situation decreased (d-1SD = –.24). These findings support the possibility of analogical-based predictive effects for real-life political events, and are discussed in light of their research and political implications.

## Introduction

On 19th July 1950, US President Harry Truman urged the Congress to deploy American troops in US-allied South Korea, which had been attacked by communist-backed North Korea a few days earlier. Truman stated in his speech:

The free nations have learned *the fateful lesson of the 1930’s*. That lesson is that aggression must be met firmly. Appeasement leads only to further aggression and ultimately to war. (Boller & Story, 1984; emphasis added)

The “lesson of the 1930’s” is a reference to the behavior of the Allies during the pre-WWII era: The Allies are often portrayed as having adopted a foreign-policy of “appeasement” in various instances of aggressions from the part of the Axis Powers (e.g., Japanese invasion of Manchuria in 1931, Italian invasion of Ethiopia in 1935), culminating in the Munich agreements of 1938. In referring to this “lesson”, Truman was equating the situation in South Korea to the pre-WWII era, suggesting that Americans ought to learn from past mistakes by doing the opposite of what the Allies did—responding to aggression with forceful aggression. Doing so would presumably lead to a better outcome than in the past.

Drawing on perceived lessons of the past to inform the present (and the future) is ubiquitous in public and political discourse (Khong, 1992; May, 1973). Policymakers often invoke such *historical analogies* to mobilize public support for a given issue (Paris, 2002). And studies indicate that mere reminders of past events can influence individuals’ representations and judgments about target issues (e.g., Gilovich, 1981; Perrott, Bodenhausen & Gentner, 2005). But does exposure to specific lessons about the past affect how people view the future? This question is particularly relevant in political contexts where historical analogies are often used to make predictions about the future—as in Truman’s example above. For such predictive effects to occur, two events—one past, one present—are matched in terms of their causal structure. If this process is successful, it should result in specific expectations about the way the current situation will turn out in the future. This hypothesis is consistent with the way research in cognitive sciences (Gentner & Forbus, 2011) and on social judgments (Read, 1983; 1984) has conceptualized analogical reasoning, as well as with the use of historical analogies in political contexts (Paris, 2002). However, it has seldom been tested in the context of ongoing, real-life events. Such events are more causally complex and ambiguous with regards to how a given lesson of the past should apply to them, than artificial or hypothetical problems often used in cognitive sciences; therefore they require empirical investigation in their own right. Accordingly, this paper presents the findings of four experimental studies which investigated the predictive effects of historical analogies in the context of ongoing real-life, political situations.

## Historical Analogies: Their uses and effects

A historical analogy is defined as “an inference that if two or more events separated in time agree in one respect, then they may also agree in another” (Khong, 1992, p. 7). The typical historical analogy consists of a source or base, which is used to draw inferences about a temporally distant event, namely the ongoing situation, or target (Gentner, 1983). Historical analogies represent a particular instance of more general analogical reasoning, which involves the following steps: (1) A source is selected and retrieved in memory, (2) common elements between the source and target are identified and mapped to one another, and (3) further similarities are inferred on the basis of those existing commonalities (Gentner & Forbus, 2011).

Political scientists have long documented the various ways in which analogies with the past are used by politicians and decision makers (see Ghilani et al., 2017). Case studies in that field (e.g., Khong, 1992) suggest that the use of historical analogies can be both a means of justifying one’s actions as well as a decision making strategy in the face of novel, uncertain and ambiguous situations. But the use of historical analogies is not restricted to politicians. Lay people seem to find meanings in them too. Political attitudes, like support for foreign military intervention, correlate with preferences for analogies with specific past events (WWII vs. the Vietnam War; Schuman & Rieger, 1992). Making such analogies experimentally available can also influence decisions made about a hypothetical conflict (Gilovich, 1981), alter the representation in memory of a target issue (e.g., Gay rights; Perrott et al., 2005), induce favorable attitudes toward minority groups (Rees, Allpress & Brown, 2013) and affect the moral evaluation of contemporary groups (Branscombe, Warner, Klar & Fernandez, 2015). Thus these studies show that comparisons with different past events can affect perceptions and judgments of a current situation, issue or groups. In doing so, analogies act in part as framing devices, organizing the information one has about the present in a way that is congruent with a familiar source (Kruglanski, Crenshaw, Post & Victoroff, 2007).

Importantly, while historical analogies seem to ostensibly compare two events, they rely in fact on specific *representations* of those events. Some events, like WWII or the Vietnam War, have specific meanings and are seen as so important to a group’s identity that their name becomes a shortcut for a range of representations, which then in turn serves political functions (Liu & Hilton, 2005). While some meanings of the past are more consensually held than others, none are fixed and unchangeable: What the past means and how it relates to the present is ultimately a matter of argument (Ghilani et al., 2020). To the extent that the past is accepted as a guide for the present, whoever manages to impose their interpretation of the former wins dominion over the latter. This is often the reason for fierce debate about historical analogies in political discourse: Different parties attempt to impose not only which past event (e.g., World War II vs. Vietnam War) should be considered as relevant to the present, but also which interpretation of that event should prevail; resulting in twin-layered “metaphor wars” (Paris, 2002). Thus, it is not just that exposure to *different* past events affects judgments about the present, but variations in the *meanings* of these events are also influential. Those meanings—or lessons—are especially important when it comes to one of the key roles played by historical analogies: their predictive power.

## Lessons of the past for the future

The idea that the past offers insights into the future is not new (Ghilani et al., 2017). In the 4th century BC, the Greek rhetorician Isocrates advised the Cyprian Prince Nicocles to “reflect on the fortunes and accidents which befall both common men and kings, for *if you are mindful of the past you will plan better for the future*” (Speech 2, section 35; emphasis added). Accordingly, historical analogies are believed to play not only *diagnostic* roles by defining a situation and its stakes, but also *prognostic* ones, by helping one assess potential courses of actions and predict their likely consequences (Khong, 1992). Both historians (May, 1973) and cognitive scientists (Indurkhya, 1992) have often deplored the logical fallacies and reasoning pitfalls involved in such “predictive analogies”, but they also acknowledge that people find them “psychologically compelling” (Indurkhya, 1992, p. 226).

Importantly, in order to make such analogy-based predictions, the two situations must be perceived as sharing key similarities in their underlying structure, as follows:

1. Situation X led to outcome Y in the past
2. Today’s situation X’ is similar to past X
3. Therefore today’s situation X’ will lead to a similar outcome Y’

This process entails matching the source and target not only in terms of their surface attributes (today’s X’ is like past X), but also in terms of their respective underlying causal structure (X is to Y what X’ is to Y’; Houghton, 1998). Note that the fourth term (Y’) has actually not taken place yet. Its future occurrence can only be predicted by assuming that the two events compared do share the same underlying causal structure.

Research on non-historical analogical reasoning has also often emphasized the importance of this kind of matching, which highlights structural similarities (Holyoak & Koh, 1987). For instance, analogies generated by laboratory participants in support of a given political point of view typically involve structural similarities, especially in terms of causal relations (Blanchette & Dunbar, 2000). Other studies found that participants relied on an analogy with a single individual for predicting the behavior of another when both individuals matched on causal features (Read, 1983; 1984).

Thus literatures in both cognitive and political sciences strongly suggest that specific processes are at play when historical analogies are used for prediction (rather than for other types of purposes). Yet the idea that reminders of the past could make some futures seem more likely than others has remained largely untested for real-life events.

## Present research

While many studies have shown how exposure to different representations of the past affects inferences drawn about a target, few have focused on the *predictive* power of historical analogies *per se* and how it derives from specific causal claims, or lessons, about the past (see Hilton et al., 1996 for correlational supporting evidence). We hypothesize that historical analogies are underlined by similar transfer processes as any other kind of analogical reasoning. If that is the case, making a variation of specific causal claims about the past available should lead to different expectations about how a current situation will turn out in the future.

We conducted four experimental studies to test this hypothesis. Participants were exposed to different “lessons” about a past event then asked about their expectations regarding the outcome of an ongoing, real-life situation. All targets constituted ongoing, major foreign-policy situations, with considerable uncertainty about their future outcomes. Such uncertainty was expected to increase reliance on analogical reasoning (Khong, 1992; Tetlock, 1998; Vertzberger, 1986). In order to avoid unpredicted motivational effects if the target was seen as too self-relevant, the situations were framed in a way that did not directly involve participants’ own countries. Specifically, studies one and two focused on the US role in the Iranian Nuclear Deal, study three on Italy’s role in the military intervention against the self-proclaimed Islamic State of Iraq and Syria (ISIS), and study four on the Catalan referendum.

### Hypotheses and overview of analyses

In all studies, we tested the main effect of exposure to different lessons about the past on predictions about target situations (Hypothesis 1).

Moreover, initially exploratory findings in Study 1 showed that such analogical effects on predictions were only present for participants with lower knowledge about the target situations they were evaluating (i.e., lower current knowledge). Previous studies on non-historical analogies had also found that individuals tend to rely on the available analogies only in the absence of a clear rule (Read, 1983). Presumably, the more knowledge one has about a current situation and/or the more familiar it appears, the more likely it is that individuals entertain stable, pre-existing views about it and can refer to their own overarching rules or schemas (Khong, 1992)—at the expense of relying on an analogy to a single exemplar. This would be in line with the notion that analogies act as heuristic tools (Tetlock, 1998; Vertzberger, 1986); with less knowledgeable and/or less motivated people relying on them more (Chaiken, 1980). Such dual effects would also fit with the Elaboration Likelihood Model of persuasion (Petty & Cacioppo, 1986), where ability to process a given message—including prior knowledge and interest for a given topic—can affect how deep (“central” vs. “peripheral”) is the perusal of said message. In light of these elements, in Studies 2-4, we expected the analogical effect on predictions to become stronger as current knowledge decreased (Hypothesis 2).

In Study 3, perceived soundness of the analogy was introduced as an additional moderator. Indeed, at its core, analogical reasoning involves the idea that elements of a source and target are comparable or similar in some respect (Gentner, 1983). As such, one would expect that the more similar two situations appear, the more likely it is that one would derive inferences from this analogy (Smeekes et al., 2014; Hypothesis 3).

All studies, except the first one, (1) were preregistered on the AsPredicted platform, and (2) used sequential analyses, which allow to “look” at the data—and potentially conclude the study—halfway through collection if results prove significant below an *a priori* defined and adjusted alpha level (Lakens, 2014). By “distributing” it across each look, the Type I error rate is thereby controlled and remains at 5% in total. In our case, halfway analyses were all inconclusive and the data collection was completed for each study in order to have more reliable estimates of the effects. Accordingly, the final analyses in studies two to four used adjusted levels of significance that were smaller than .05 (though the overall Type I error over the entire data collection still amounts to 5%).

Moreover, despite our subtle manipulations and in order to control for potential demand effects, participants were asked at the end of each study what were the hypotheses we were trying to test, and we coded their open answers. While 4% to 44% of participants depending on the study found the true purpose of the manipulation (or mentioned something close enough), results did not change when controlling for this variable (see *Supplementary Material*). Thus, only analyses without this covariate are reported in this paper.

## Availability of data, code and material

All material, data (including the entire list of collected variables not reported here), code for reproducing the analyses and preregistrations protocols (for Studies 2–4) are provided *online* on the Open Science Framework.

## Studies 1 and 2

In the first two studies, which constituted exact replications of one another, we tested the predictive effect of historical analogies by making available different causal antecedents for the outbreak of WWII (the 1919 Versailles Treaty vs. The 1938 Munich Agreement) and manipulating whether a similar causal antecedent was present in the target situation, namely the negotiations surrounding the 2015 Iranian Nuclear Deal. This experimental paradigm reproduced in a subtler fashion the way historical analogies—including with Versailles and Munich—had been used by the media in the context of the Nuclear Deal to advocate for a more or less lenient stance toward Iran (e.g., Woodruff, 2015).

Thus, this was a 2 (WWII Lesson: Versailles, Munich) × 2 (US Policy: Uncompromising, Conciliatory), between-subjects design. We predicted that the presence of similar causal antecedents in both the past and present cases would affect predictions about future outcomes. Participants should expect more negative future outcomes when they are told that the US has adopted the same stance against Iran as European leaders did against Germany prior to WWII (i.e., being uncompromising like in 1919 Versailles vs. conciliatory as in 1938 Munich) than when told the US have adopted the opposite stance to past European leaders. In other words, participants should expect that “history will repeat itself” (i.e., by resulting in negative future outcomes) to the extent that similar past policies are pursued in the present. We tested this in two studies, one conducted in March 2015 in a Belgian undergraduate sample and the other—a preregistered replication—in August 2016 among British citizens of various backgrounds. Except for minor adjustments to account for language and context differences, the design was virtually identical in both studies.

### Sampling and Participants

In Study 1, an *a priori* power analysis determined that for a 2 × 2, between-subjects ANOVA, assuming a medium sized effect (*f* = .25) and a desired power of .80, the minimum sample size needed was 128. After excluding 24 participants who did not complete the questionnaire, paused too long midway through and/or failed all attention checks, the final sample consisted of 132—mostly Belgian—undergraduate psychology students (29 males), with a mean age of 20.44 (*SD* = 2.87), who took part in the study in exchange for course credits.

In Study 2, we used a sequential analysis (Lakens, 2014). We planned one interim analysis halfway through data collection and, if the analyses proved non-significant at that stage, to complete the collection (final alpha = .0336; see preregistration protocol *online*). Participants were recruited online via the crowdsourcing platform Prolific Academic and the questionnaire was restricted to native English-speakers, who were UK-born and older than 18-year-old. At the interim analysis (*N* = 166), the planned analyses were not significant at the adjusted alpha level so the data collection was carried out to its term. In keeping with the preregistration, we excluded a priori 33 individuals who failed various attention checks and/or did not complete the entire questionnaire. The final sample size of study 2 consisted of 392 participants (44% males) with a mean age of 34.54 (*SD* = 12.23). All were English-speaking individuals, living in the UK and had completed the questionnaire in exchange for financial compensation. Most were employees (61%) or students (18%), and a majority (96%) held at least a high school degree.

### Procedure

Participants in both studies completed an online questionnaire and were randomly assigned to one of the four conditions. The questionnaire consisted of two parts, containing respectively the manipulation of the source and the target. In order to decrease demand effects, each part was presented as independent studies with their own cover story. Participants were told the lesson exposure stage was to help us evaluate the historical texts we would use in future studies, while the target manipulation stage was meant to gather their perceptions about news events. In the lesson exposure stage, participants read either a text describing the Versailles Treaty or the Munich Agreement, as well as their respective “lesson” (that “harsh” treatment of Germany in Versailles vs. “appeasement” of Nazi Germany in Munich was instrumental in the occurrence of WWII).

In the subsequent target manipulation stage, we gave participants a (fake) newspaper article allegedly originating from “Le Monde Diplomatique” (Study 1) or “The Guardian” (Study 2). The article described the context of Iran’s nuclear development and the Nuclear Deal. Negotiations about the Deal were ongoing at the time of study 1 in March 2015 but had been completed for a year at the time of study 2 in August 2016 (Resolution 2231; United Nations, 2015). Adjustments to the material in study 2 accounted for this contextual change, but in both cases the Iranian Deal (either forthcoming or already formalized) was described as the product of either an uncompromising or conciliatory stance on the part of the US (see exact wordings *online*). After reading the article, we measured participants’ expectations about different future outcomes following the signing of the Deal. In study 2, this included items on perceived future inevitability and foreseeability. After completing the questionnaire, participants were thanked and later debriefed (collectively at the lab in study 1 and via email in study 2).

### Measures

Unless otherwise noted, all variables were measured on 7-points, Likert-type scales (see ***Table 1*** for descriptive statistics).

**Table 1:** Mean and standard deviations of the main variables by experimental condition in Studies 1 and Study 2.


	STUDY	MUNICH – US CONCILIATORY	MUNICH – US UNCOMPROMISING	VERSAILLES – US CONCILIATORY	VERSAILLES – US UNCOMPROMISING

		N_1_ = 31/N_2_ = 112	N_1_ = 34/N_2_ = 79	N_1_ = 33/N_2_ = 110	N_1_ = 34/N_2_ = 91

Expectations about Iran’s future behavior	1	–11.77 (43.76)	–8.09 (45.56)	7.70 (47.68)	–12.06 (37.64)

	2	13.93 (39.23)	7.13 (40.6)	21.22 (39.35)	14.81 (35.94)

Approval for US policy	1	3.74 (1.22)	3.91 (.98)	4.11 (1.19)	3.97 (1.02)

	2	–	–	–	–

Inevitability of future negative outcome	1	–	–	–	–

	2	3.47 (1.41)	3.44 (1.13)	3.20 (1.34)	3.46 (1.28)

Foreseeability of future negative outcome	1	–	–	–	–

	2	2.99 (1.45)	2.82 (1.38)	2.87 (1.48)	2.97 (1.37)

Current Knowledge	1	4.05 (1.16)	3.83 (1.27)	3.56 (1.23)	3.53 (1.18)

	2	3.86 (1.07)	3.99 (1.09)	4.13 (1.08)	3.88 (.94)


*Note*: Standard deviations appear in parentheses. For the 1^st^ variable (Expectations about future outcomes), the scale ranges from –100 to 100 (a more positive score = higher expectations for the positive future outcome relative to the negative one). All other scales are 7-points Likert-type ones. Cells containing dashes indicate that the corresponding variable was not measured in that study. n_1_ = sample size for study 1; n_2_ = sample size for study 2. Depending on the condition, past knowledge related to the Munich Accord or to the Versailles Treaty.

*Expectations about Iran’s future behavior* (main dependent variable) were assessed by asking participants the likelihood of three possible courses of action that the Iranian government might pursue within five years, following the US negotiation stance. Each estimate could range from 0% (not at all likely) to 100% (totally certain), but all three estimates had to sum up to a 100% (adapted from Fischhoff, 1975). The outcomes depicted either a positive nuclear energy application (“Respect the terms of the international community and use its nuclear energy for a civilian purpose; e.g., energetic production”), negative (“Keep enriching its uranium and use its nuclear program for a military purpose; e.g., weaponry”), or a relatively neutral application (i.e., “Totally stop its nuclear program and develop another kind of energetic resource”). The final DV consisted of the difference in estimates between the positive and negative outcomes.

*Approval for US negotiation style during the Iranian Deal* (DV – study 1 only) was the average of two items (“How much do you approve of the type of approach adopted by the US toward Iran?” and “How much do you expect this current US policy will avoid a military use of nuclear energy by Iran”; r = .39, p < .001).

*Perceptions of inevitability and foreseeability of a future negative outcome* (DVs – study 2 only; adapted from Nestler et al., 2010) were included to see if the manipulation affected other future-related measures than specific expectations about future outcomes. Seven items measured perceived inevitability (e.g., “Following the Iranian deal, the military use of nuclear energy by Iran in the future is inevitable”; α = .87) and four items measured foreseeability of the negative outcome (e.g., “It is perfectly clear to me that signing the Iranian deal will lead to a military use of nuclear energy by Iran”; α = .82).

*Current knowledge* (moderator – study 1: α = .83; study 2: α = .81) was measured via six items tapping into reported knowledge and interest about general news and about the Iranian nuclear program in particular (e.g., “I rarely keep informed about the news”, “how would you rate the extent of your knowledge regarding Iran’s Nuclear program?”; from 1 not at all/very low to 7 absolutely/very high).

*Past Knowledge* (exploratory measure). Knowledge about the Versailles Treaty or the Munich Agreement (depending on the condition) was measured on a single item right after participants read the text describing one of the two past events (“how would you evaluate your level of knowledge about the event described [i.e., Versailles or Munich]?”, from 1 *very low* to 7 *very high*).

## Results and Discussion

### Study 1

#### Main analyses

A 2 × 2, between-subject ANOVA tested the interaction effect of WWII Lesson × US Policy on the main dependent variable—expectations about Iran’s future behavior. It was not significant, F(1, 128) = 2.36, p = .127, η_p_^2^ = .018. A separate ANOVA yielded no effect either on approval for the US approach toward Iran (F(1,128) = .626, p = .430, η_p_^2^ = 005). Thus, the expected interaction effect was not found on either dependent variable.

#### Exploratory Analyses

**Figure 1:**
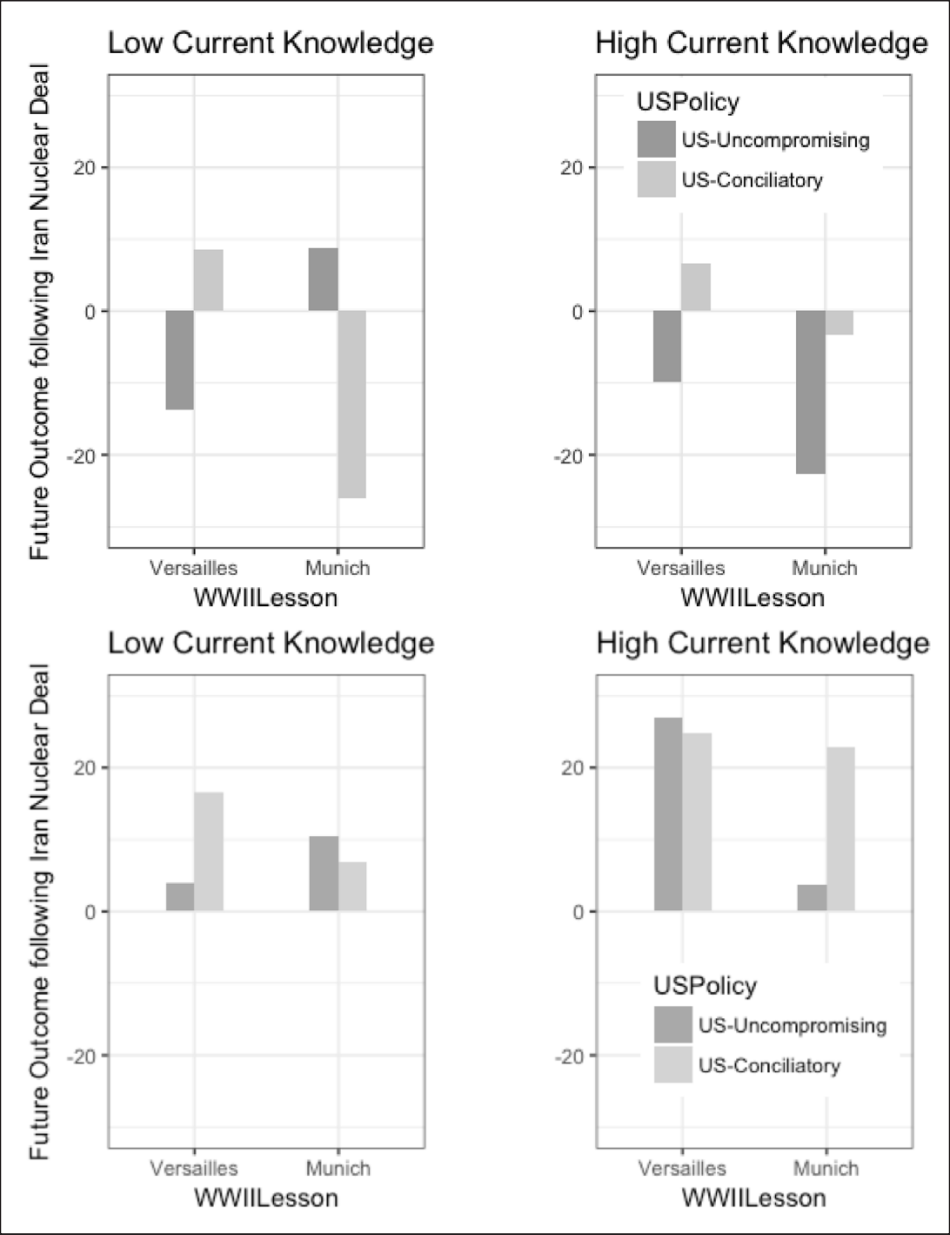
In Study 1 (top row) and Study 2 (bottom row), expectations that Iran will use its nuclear energy for civilian rather than military purposes in the future (on a scale from minus 100% = military purpose, to plus 100% = civilian purpose), depending on the “historical lesson” participants were reminded of (Versailles vs. Munich) and the current policy they believed the US had adopted toward Iran (Conciliatory vs. Uncompromising). Results of the moderation by current knowledge appear respectively on the left (–1 standard deviation) and on the right (1 standard deviation).

We decided to explore the dataset further to reveal potential moderators. We ran 2 × 2 ANOVA tests using the knowledge-related measures as dependent variables to ensure that they were not affected by the manipulation. The variables were then standardized and entered in model 3 of Hayes’ (2012) SPSS-implemented, Process matrix (version 2.11) to test for moderated moderation effects. While past knowledge did not seem to play a role in the analogical effect (see *Appendix D*), we found a marginally significant, three-way interaction between WWII lesson × US Policy × Current knowledge (continuous moderator) on expectations about Iran’s future behavior (β = .17, t = 1.91, p = .058; 95% [–.01, .35]). Simple effects at one plus and minus the SD of current knowledge yielded a significant first-order interaction effect only at minus 1 SD (β = –.32, t = –2.67, p = .008; 95% [–.56, –.08]; see *Appendix A* for detailed simple effects). Hence, the hypothesized pattern was obtained for those who tended to report lower levels of current knowledge (see ***Figure 1***, left side). However, those with higher current knowledge tended to disregard the available lesson for their judgments (β = .01, t = .13, p = .899; 95% [–.25, .28]; see ***Figure 1***, right side). No such moderating effect was found on the second DV (see ***Table 2***; see alternative, higher-powered analysis in *Appendix B*). These results were promising but resulted from exploratory (rather than confirmatory) analyses, enhancing the risk of Type 1 error. Therefore, we attempted to replicate them in a second preregistered study, with a more diverse and higher-powered sample.

**Table 2:** Effect of the experimental manipulation (WWII Lesson ⋅ US Policy) and the moderation by Current Knowledge on the dependent variables in Studies 1 and 2.


	EXPECTATIONS ABOUT FUTURE OUTCOMES						APPROVAL FOR US POLICY			PERCEIVED FORESEEABILITY OF FUTURE OUTCOME			PERCEIVED INEVITABILITY OF FUTURE OUTCOME		
			
	STUDY 1			STUDY 2			STUDY 1 ONLY			STUDY 2 ONLY			STUDY 2 ONLY		
				
	*β*	*T*	*P*	*β*	*T*	*P*	*β*	*T*	*P*	*β*	*T*	*P*	*β*	*T*	*P*

Intercept	–.01	–.11	.915	.01	.12	.901	.00	.02	.986	–.01	–.12	.904	–.00	–.09	.928

Current Knowledge	–.02	–.26	.790	.13	2.75	**.006**	–.07	–.68	.498	–.03	–.53	.593	–.09	–1.74	.081

WWII Lesson	–.09	–1.03	.303	–.09	–1.77	.078	–.09	.92	.358	–.03	–.55	.580	.04	.85	.393

US Policy	.07	.74	.460	.08	1.61	.109	–.01	–.11	.922	–.00	–.09	.926	–.04	–.85	.393

Lesson × Policy	–.15	–1.68	.096	.01	.32	.744	–.07	–.78	.435	.04	.86	.389	.04	.88	.380

Lesson × Current Knowledge	–.04	–.41	.679	–.05	–1.14	.271	–.05	–.49	.626	–.01	–.30	.762	.02	.34	.732

Policy × Current Knowledge	.13	1.50	.135	.02	.46	.647	.06	.65	.519	–.02	–.36	.719	–.06	–1.25	.213

Lesson × Policy × Current Knowledge	.17	1.91	.058	.12	2.47	**.014**	.05	.47	.638	–.04	–.81	.417	–.06	–1.13	.258


*Note*: Results obtained using Model 3 of Process (Hayes, 2012). Alpha level in Study 1 = .05, Study 2 = .0336; p values below alpha level are indicated in bold font. Sample sizes: N_Study1_ = 132; final N_Study2_ = 392. The dependent variables used in each model appear in columns (only the 1^st^ DV appears in both studies 1 & 2; the rest was only measured in one of the two). All variables are standardized.

### Study 2

#### Preregistered Analyses (alpha level = .0336)

The WWII Lesson × US Policy interaction was tested using Model 1 in Process. Results showed no effect on either dependent variable (see ***Table 2***). However, and in line with our hypothesis, when current knowledge was included in the model as a continuous moderator, the three-way interaction was significant at the adjusted alpha level (β = .13, t = 2.47, p = .014; 95% [.02, .22]). An analysis of simple effects indicated that the difference was in the expected direction. As in Study 1, individuals with lower current knowledge tended to have more analogy-congruent expectations (β = –.11, t = –1.61, p = .108; 95% [–.24, .02]). However, better-informed participants tended to neglect the analogy when making their predictions (β = .14, t = 1.95, p = .052; 95% [–.00, .29]). In the Munich condition specifically, their predictions were in fact exactly the reverse of what the lesson would suggest (***Figure 1***, bottom row; see *Appendix A* for detailed simple effects & *Appendix B* for alternative analysis with combined experimental conditions).

These findings are consistent with our hypothesis that exposure to specific lessons of the past affects predictions made about a current situation, but this effect seems to depend on the extent of current—and not past—knowledge. Moreover, this interaction effect was only found on the same measure used in study 1 (expectations about future outcomes) and not on the other two dependent variables (see ***Table 2***)—a point we will return to in the general discussion.

In summary, these two studies show that the same political stance (e.g., being conciliatory toward Iran) was expected to lead to more negative outcomes when participants had been exposed to the Munich lesson vs. the Versailles lesson. However, this predictive effect tended to emerge only as participants’ current knowledge decreased. This pattern, found in study 1 in a relatively small sample of Belgian psychology undergraduates, was replicated in study 2, in a larger sample of more demographically diverse UK participants. Moreover, study 2 suggested it was unlikely that this effect was solely related to differences in morality appraisals elicited by the Versailles vs. Munich lessons (see *Appendix B*). However, it was possible that the predictive effect resulted from differences in some other features between the Versailles and Munich analogies and/or that participants had varied in their expectations about Iran prior to the manipulation—a possibility that our between-subject design could not rule out. The third study addressed both limitations by (1) highlighting the same past antecedent and outcome in all conditions, and changing only how their respective relation was construed—thus controlling potential confounds, and (2) by using a within-subject design to assess the change in participants’ expectations after exposure to a historical analogy while controlling for their initial (i.e., pre-manipulation) expectations.

## Study 3

The target situation consisted of the multinational coalition effort put in place to fight the self-proclaimed Islamic State of Iraq and Syria (ISIS)—and more specifically, the part played by the Italian government in the coalition. Whether the coalition would be successful in destroying ISIS and their ability to carry out terrorist attacks in the future was an open—and much debated—question at the time. We decided to use an analogy with a past US military intervention—Operation Infinite Reach—conducted in 1998 against Al Qaeda targets. After US embassies in Kenya and Tanzania were bombed in attacks attributed to Al Qaeda, President Bill Clinton had ordered airstrikes against Al-Qaeda training camps in Afghanistan and against a Sudanese pharmaceutical factory believed to be manufacturing chemical weapons for Osama bin Laden—an intelligence that was later questioned (Lacey, 2005). This Operation had the particularity of being the last, and only, direct US military intervention against Al Qaeda targets prior to the September 11th, 2001 attack (9/11). We manipulated the perceived causal link between this Operation and the 9/11 attack. Our purpose was to see if expectations about the current anti-ISIS intervention would change if participants were told that the 1998 Operation either had directly caused the 9/11 attack (as retaliation) vs. that it had managed to prevent a worse, long-planned attack.

### Design and hypotheses

The questionnaire consisted of three stages: (1) A pre-manipulation stage where participants read about the target situation and completed the baseline measures including their knowledge about the situation described, their expectations about how successful the intervention will be in eliminating ISIS forces (DV1) and the perceived likelihood of a future terrorist attack in Italy (DV2). Step (2) of the study was the lesson exposure stage: A (fake) newspaper article describing the 1998 Operation Infinite Reach was presented and participants were told it had been randomly selected by the software in a large database of terrorism-related articles. The article, titled “Operation Infinite Reach: 18 years later”, contained a brief description of the operation: its motivations (the attacks on US embassies in Kenya and Tanzania attributed to Al-Qaeda), its targets (the Afghan camps where bin Laden was expected to be found and the pharmaceutical factory believed to be manufacturing chemical weapons for Al-Qaeda), and its chronological antecedence to 9-11 (the fact that it was one of the last military actions conducted by the US against Al-Qaeda prior to 9/11). To that point, the article was identical in both conditions. Then one last paragraph was included which purported that recently uncovered evidence demonstrated either that the 1998 Operation had caused the 9/11 attacks in an act of retaliation for killing some of bin Laden’s family members (Operation-caused-9/11 condition), or that the 9/11 attacks had been planned long before the 1998 Operation and that the latter had actually been successful in preventing what would have been a worse attack (Operation-prevented-worse-attack condition). After reading this text and answering a few questions about it (including some attention checks), participants entered the third, post-manipulation stage of the questionnaire. They were told that, in order to investigate the link between short-term memory, attention span and the perception of complex situations, they would be again asked questions regarding the first article presented (i.e., about the current Italian intervention). The dependent and moderating variables were collected in that stage.

Hence, this was a 2-conditions, between-subjects design, with the pre-manipulation measures used as covariates. Three hypotheses were preregistered: Participants in the Operation-caused-9/11 condition should expect the current Italian intervention to be less successful, and a terrorist attack on Italian soil more likely, compared to those in the Operation-prevented-worse-attack condition (H1). Based on the two previous studies, we expected that these effects would become stronger as current knowledge decreased (H2). Finally, given that the exposure to the analogy was more explicit than in the previous two studies, we also predicted that the main effect would be stronger the greater the perceived soundness of the analogy between the current Italian intervention and the 1998 Operation (H3).

### Participants and Procedure

A sequential analysis with one look halfway through the data collection was planned (see preregistered protocol *online*), but the interim analysis was non-significant and the other half of the sample was therefore collected. The study was conducted online from late November 2016 until mid-April 2017. The sample was collected in three ways: Among psychology undergraduate students in exchange for course credits (41%), via crowdsourcing platforms in exchange for payment (29%) and through the personal networks of the experimenters (30%). Participants were randomly distributed between the two experimental conditions and were told that the general aim of the study was to better understand how individuals made sense of complex social situations. After completing the questionnaire, participants were thanked and later received a written debriefing disclosing the real purpose of the study.

As preregistered, we excluded *a priori* participants who did not complete the questionnaire, missed various attention checks, and/or exhibited suspicious IP addresses and reaction times (see *online* data for details). Although it was not mentioned in the preregistration, we also excluded 10 participants of Italian origin by fear that the self-relevance of the target situation may affect their answers (though we ran the analyses after including those participants in the sample and results did not significantly change). In total, 57 participants were excluded and the final sample size consisted of 361—mostly Belgians and French—individuals (36% males), with a mean age of 28.36 (*SD* = 12.59); most of whom were either students (57%) or employees (25%).

### Measures

Unless otherwise noted, all items were measured on 7-points, Likert-type scales.

#### Pre-manipulation measures (covariates).

*Initial expectations about the outcome of the Italian intervention* was measured via one item “These military strikes, including Italian ones, will manage to annihilate the biggest part of ISIS’ forces”. This item was presented amid other measures tapping into the perceived legitimacy of the Italian intervention but not used in the confirmatory analyses.

*Initial expectations about a future terrorist attack in Italy* was assessed via five items averaged in a single scale (e.g., “It is probable that following its military intervention, Italy will soon be the target of retaliation on the part of ISIS”; α = .72).

#### Dependent variables (post-manipulation).

The main DV, *expectations regarding the outcome of the Italian intervention*, was measured as in the two previous studies by rating on a scale from 0% to 100% the perceived likelihood that the Italian intervention will result in three outcomes within two years’ time: positive (“The Italian intervention will be successful and will strongly reduce ISIS’ power”), negative (“The Italian intervention will reinforce ISIS’ determination and its expansionist ambitions”) and neutral (“The Italian intervention will have no impact on ISIS”). The sum of the estimates had to amount to 100%. This DV correlated significantly with the corresponding pre-manipulation measure (r = .35, p < .001).

*Expectation about a future terrorist attack in Italy* following its role in the anti-ISIS intervention was assessed via a single item ranging from 0% (certainly not) to 100% (certainly). This second DV correlated significantly with the corresponding pre-manipulation measure (r = .62, p < .001).

*Confidence in predictions* regarding both the success of the Italian intervention (“To what extent do you feel [uncertain/assured/hesitant] about your answers”; 3 items), and a future terrorist attack (“To what extent are you certain about your answer”; 1 item) were aggregated in a single scale (α = .78).

### Moderators

*Current Knowledge*. Before reading the text about the Italian intervention, participants were asked to report their level of general knowledge about that event on a single item. After reading the text, they were asked again via three items the extent to which the information given appeared new, familiar (reverse scored) and well-known (reverse scored). Finally, at the end of the questionnaire, four items (the same used in the two previous studies) tapped into the level of information and interest for the news in general. An average score was computed out of these eight items (α = .76).

*Perceived soundness of the analogy* between the US 1998 Operation against Al-Qaeda and the current Italian intervention against ISIS was assessed via four items (e.g., “The current Italian intervention looks like the military strikes conducted in 1998 by the US”), averaged in a single score (α = .83).

*Past knowledge* (about the 1998 Operation Infinite Reach – Exploratory measure) was measured via a single item (“how they would estimate their level of knowledge about the 1998 Operation described in the article”; from 1 *I had never heard of it* to 7 *I am very well-informed*).

All variables were standardized prior to the analyses and descriptive statistics appear in ***Table 3***.

**Table 3:** Means (and standard deviations) of the main variables in Study 3 (total N = 361).


	OPERATION CAUSED 9/11 CONDITION	OPERATION PREVENTED WORSE ATTACK CONDITION

	N = 195	N = 166

Baseline expectation that Italian intervention will be successful (covariate 1)	3.20 (1.59)	3.3 (1.61)

Baseline expectation that a terrorist attack will occur in Italy (covariate 2)	4.49 (1.07)	4.79 (.93)

Expectations about future success of Italian intervention (DV1)	–4.89 (31.91)	–13.07 (32.26)

Expectations about future terrorist attack in Italy (DV2)	6.84 (1.86)	7.09 (1.74)

Current Knowledge (moderator 1)	3.51 (.96)	3.55 (1.08)

Confidence in predictions	3.85 (1.34)	3.88 (1.22)

Endorsement of the analogy between 1998 US intervention & Italian intervention against ISIS (moderator 2)	3.83 (1.39)	3.94 (1.29)


*Note*: Standard deviations appear in parentheses. Expectations about future outcomes (DV1) range from –100 to 100 (a more positive score = higher expectations for the positive future outcome relative to the negative one) and the likelihood of a future terrorist attack in Italy (DV2) ranges from 1 (0% certainly not) to 11 (100% certainly). All other variables were measured on 7-points, Likert-type scales, where higher number = higher expectations/current & past knowledge/analogy endorsement/confidence.

## Results and Discussion

### Main effect of analogical manipulation (hypothesis 1)

The main effect of the lesson manipulation on expectations about the success of the Italian intervention was tested in an ANCOVA, with the corresponding pre-manipulation measure as a covariate. Results indicated a significant effect of the condition at the adjusted alpha level of .0336 and in the expected direction (*F*(1,358) = 7.77, p = .006, η_p_^2^= .021) : Participants in the Operation-caused-9/11 condition expected the current Italian intervention to be less successful in reducing ISIS’ power (adjusted M = –9.59, SD = 33.75) than those in the Operation-prevented-worse-attack condition (adjusted M = –5.30, SD = 31.26^1^). A separate ANCOVA tested the main effect of the manipulation on the second DV—i.e., expectations about a future terrorist attack in Italy (using its corresponding pre-manipulation measure as covariate). However, the result on that DV was not significant (F(1,358) = .29, p = .590, η_p_^2^ < .001). Thus, the first hypothesis was supported, though only in the case of the first DV. After ensuring that there was no difference between the experimental conditions on the two moderators, the two moderation hypotheses were tested via Model 1 in Process.

### Moderation by current knowledge (hypothesis 2)

**Figure 2:**
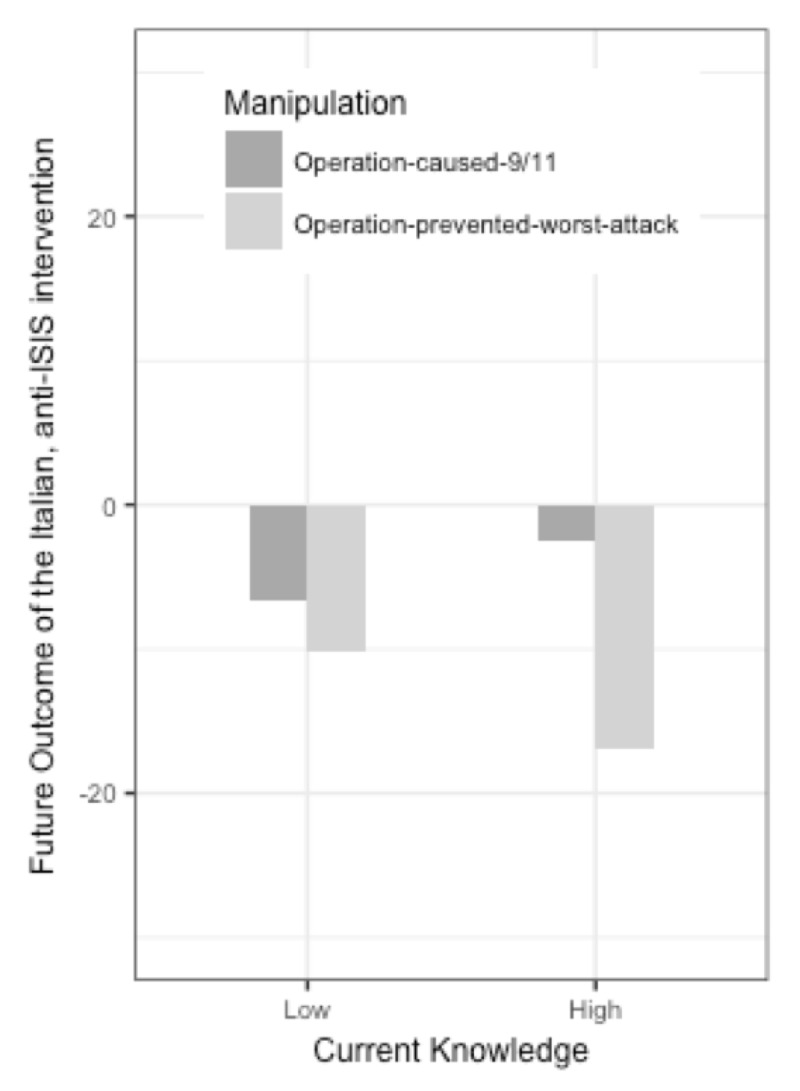
In Study 3, expectations that the Italian intervention against ISIS will be successful in the future (on a scale from minus 100% = not successful and will increase ISIS’ expansionist ambitions, to plus 100% = highly successful in diminishing ISIS’ power), depending on the “historical lesson” made accessible to participants (i.e., that there was a strong causal link in the past between the 1998 US intervention and the 9-11 attack vs. no such link). Results of the moderation by current knowledge appear respectively on the left (-1 standard deviation) and on the right (1 standard deviation).

The lesson × current knowledge interaction on expectations about the success of the Italian intervention (controlling for the corresponding pre-manipulation measure) was not significant (β = .08, t = 1.64, p = .101; 95% [–.02, .18])—although the pattern of simple effects was in the expected direction: Participants with lower current knowledge tended to have more analogy-congruent expectations (β = –.22, t = –3.37, p = .001; 95% [–.35, –.09]) than those with higher current knowledge (β = –.05, t = –.73, p = .467; 95% [–.20, .09]; see ***Figure 2***). The moderation effect was not significant when using expectations of future terrorist attack in Italy as DV (β < .00, t = .01, p = .987; see ***Table 4***).

**Table 4:** Main and moderation effects on the dependent variables of Study 3.


	EXPECTATIONS ABOUT FUTURE SUCCESS OF ITALIAN INTERVENTION			EXPECTATIONS ABOUT A FUTURE TERRORIST ATTACK IN ITALY		
	
	β	T	P	β	T	P

Intercept	–.00	–.03	.977	–.00	–.00	.999

Current Knowledge	.01	.25	.799	.06	1.36	.173

Covariate (corresponding baseline measure)	.34	5.94	**<.001**	.62	15.83	**<.001**

Experimental condition	–.14	–2.81	**.005**	–.02	–.54	.587

Experimental condition × Current Knowledge	.08	1.64	.101	.00	.01	.987

Intercept	–.00	–.03	.973	–.00	–.01	.991

Analogy endorsement	–.22	–4.58	**<.001**	.13	3.25	**.001**

Covariate (corresponding baseline measure)	.35	6.31	**<.001**	.62	15.72	**<.001**

Experimental condition	–.13	–2.68	**.008**	–.03	–.65	.515

Experimental condition × Analogy endorsement	.04	.81	.421	.01	.28	.781


*Note*: These results were obtained using Model 1 of Process (Hayes, 2012). Alpha level = .0336; p values below this alpha level are indicated in bold font. Total N = 361. Experimental condition = either being told that the 1998 US intervention caused the September 11th 2001 attack *vs*. that 1998 US intervention prevented a worse attack; Analogy endorsement = endorsement of the analogy between the 1998 US intervention against Al-Qaeda and the 2017 Italian intervention against ISIS.

### Moderation by perceived soundness of the analogy (hypothesis 3)

The extent to which participants explicitly endorsed the analogy between the current Italian intervention and the past US one did not moderate the effect of the manipulation on either dependent variables (see ***Table 4***). While this ran contrary to our third hypothesis, it is in keeping with what was found in Studies 1–2. The hypothesis had been included here because we expected the analogical manipulation to be more obvious to participants, but only very few (N = 13) seem to have discovered the true purpose of the study and re-running the analyses after excluding these participants did not affect the findings (see *Appendix F*).

A closer look at the correlations between the main variables in the study indicated that participants with higher current knowledge tended to have more knowledge about the 1998 Operation (r = .27, p < .001) and to be more confident in their predictions (r = .17, p = .001)—a correlational pattern similar to the one obtained in the two previous studies.

In summary, participants who were told that a past US military intervention against Al-Qaeda had been causally instrumental in the occurrence of the 9–11 attack expected a significantly more negative outcome following the current Italian intervention against ISIS, compared to those who were led to believe in the opposite causal claim (i.e., that the past intervention had been effective in preventing a worse attack). Contrary to Studies 1–2, this effect was found for all participants—regardless of their current knowledge. And just like previous studies, it was also found regardless of how much participants explicitly endorsed the analogy between the two situations (i.e., the anti-ISIS and the anti-Al Qaeda intervention).

Thus this third study lends further support to the notion that exposure to lessons of the past influences people’s expectations about an ongoing, real-life situation. It could be argued that the manipulation in this study involved learning a general rule regarding the behavior of a particular group (terrorist organization). Yet, the fact that such a rule was “learned” from the specific case at hand (the 1998 US intervention against Al-Qaeda) is still an analogically-based inference involving learning a lesson from a past event and applying it to the present (the 2015 Italian intervention against ISIL)—just like learning about Germany’s behavior after Munich or Versailles and applying it to current US-Iran relations in Studies 1 and 2. Indeed, what distinguishes analogical reasoning from other types of rule-based reasoning is that it involves reasoning from one or more specific cases—even if the sum of that learning can be later abstracted into a more general rule or schema (Gick & Holyoak, 1983).

In particular, this influence seems directly related to the availability of specific causal claims about a past event and its outcome. If this is true, then reducing the perceived *importance* of a given antecedent in bringing about a past outcome—without changing any of the events in the sequence—should weaken the predictive effect of analogies. In other words, if the past event is not seen as a major, diagnostic cause for the occurrence of a past outcome, then it is less likely to be relied on for making predictions in a present situation. A fourth study tested this possibility while adding an “objective” measure of current knowledge (i.e., true/false questions about the target situation) alongside the self-reported measure used in the three previous studies.

## Study 4

Lessons of the past, and their causal claims, often rely on implicit counterfactuals about the past. For instance, the Munich analogy used in the first two studies about the “failure” of appeasement carries with it the counterfactual that *if* France and Britain had stood firm against Hitler’s demands in 1938, the course of History would have been different. This counterfactual is then applied to the present case via a counterfactual inference (Brändström et al., 2004). In all previous studies, the lesson manipulation relied on such implicit counterfactuals that *reinforced* the perceived causal link between the past antecedent and a given outcome. However, counterfactuals can also be used to *undermine* a causal link and introduce a sense of contingency (Roese & Olson, 1996). Thus, in this fourth study, we manipulated the causal claim about the past by changing what alternatives participants came to envision about it, to create the sense that the focal antecedent had played either a major vs. a minor role in bringing about the past outcome.

The target situation related to the aftermath of the referendum for self-determination held by the Spanish autonomous region of Catalonia in October 2017. Deemed anti-constitutional by the Spanish government and boycotted by anti-independentist parties in Catalonia, the referendum resulted in a majority of voices in favor for self-determination—though attendance was relatively low. A few weeks later, the Catalan parliament unilaterally declared its independence, prompting the Spanish senate to suspend Catalonia’s autonomous status and dissolve its parliament. The situation elicited parallels with past events—in particular to the break-up of Yugoslavia and Kosovo’s fight for independence (e.g., Reuters, 2017). This analogy seemed to offer a potential ground for testing our hypotheses, since Kosovo had also undergone a referendum for self-determination followed by a unilateral declaration of independence in 1991. By manipulating the extent to which this referendum appeared as a major (vs. minor) cause in the escalation of violence and subsequent Kosovo War, we could see whether expectations regarding the future of post-referendum Catalonia would grow relatively bleaker in consequence.

### Design & Hypotheses

An identical design as in Study 3 was used, with pre- and post-manipulation measures of expectations regarding the future outcome of the Catalan referendum. The only difference was the inclusion of true/false questions right at the start of the questionnaire to measure participant’s objective knowledge about the Catalan referendum before reading a description of the situation (see detailed measures in *Appendix E*). Then participants read a second (analogical source) text titled “The Kosovo, 25 years later”, which described the Kosovo situation as an autonomous region within ex-Yugoslavia, its 1991 referendum for independence followed by the escalation of violence during the Kosovo War and ending with its constitution as an independent state in 2008 (inspired from Fierstein, 2008). The Kosovo text was identical in both conditions except for one paragraph (depending on the condition, the italicized segment was replaced by the segment in brackets):

According to some specialists of the Balkans, it is clear that the independence push of the Kosovars has likely *been the main trigger for the tensions that followed. If attempts at dialogue and compromise had been put in place rather than a unilateral declaration of independence, the escalation of violence would likely not have occurred* (**major causal role condition**) [played but a minor role in the tensions that followed. Even if the referendum had not taken place, a set of regional events (e.g., the fall of the Soviet bloc and the election of Serbian nationalist Milosevic) would still have led to the escalation of violence (**minor causal role condition**)].

Finally, dependent variables were collected in the post-manipulation stage (see *Appendix E*). Thus, this was a 2-conditions (causal role: major, minor), between-subjects design, with the pre-manipulation measure treated as covariate in the analyses. As in previous studies, we expected a main effect of the causality manipulation on both DV’s (H1) and a moderation effect by current knowledge (H2).

### Sampling & Participants

As in previous studies, a sequential analysis with one “look” halfway through the data collection was planned beforehand (see preregistration *online*) but results proved non-significant at that point so we completed the data collection. Participants were recruited online via social media (32%) and among psychology undergraduate students in exchange for course credits (68%). They were told the purpose of the study was to understand how people made sense of complex social situations and assess the variables involved in text comprehension. After excluding 49 participants who failed various attention checks, were younger than 18 years old and/or were of Spanish, Kosovar or Serbian nationality, the final sample consisted of 397—mostly Belgian (74.6%)—participants (92 males) with a mean age of 25.02 (SD = 12.68). Most were in possession of a high school degree or higher (94.7%) and were currently students (80.6%).

## Results and Discussion

The preregistered analyses were run (one-tailed tests with an alpha boundary of .0354) but no significant main or interaction effects were detected on any dependent variable (see ***Table 5*** for a summary and *Appendix E* for detailed results).

**Table 5:** Main and moderation effects on the dependent variables of Study 4.


	EXPECTATIONS ABOUT POSITIVE FUTURE IN CATALONIA			APPROVAL FOR FUTURE INVESTMENT IN CATALONIA		
	
	*β*	*T*	*P*	*β*	*T*	*P*

Intercept	–.00	–.00	.998	.00	.01	.993

Self-reported Current Knowledge	.10	2.30	.022	–.04	–.86	.390

Covariate (corresponding baseline measure)	.46	10.49	**<.001**	.19	3.81	**<.001**

Causal role manipulation	.02	.55	.583	.06	1.27	.205

Causal role × Self-reported Current Knowledge	–.02	–.48	.631	.07	1.48	.138

Intercept	.00	.00	.998	.00	.00	.992

Objective Current Knowledge	–.06	-1.37	.392	–.07	-1.52	.129

Covariate (corresponding baseline measure)	.48	10.81	**<.001**	.18	3.69	**<.001**

Causal role manipulation	.02	.54	.586	.06	1.29	.197

Causal role × Objective Current Knowledge	–.01	–.25	.805	–.04	–.89	.372


*Note*: These results were obtained using Model 1 of Process (Hayes, 2012). Alpha level = .0336; p values below this alpha level are indicated in bold font. Total N = 397. The p values are reported here in **bilateral**, but according to the preregistration of this study, one-tailed tests were conducted (i.e., the reported p values in the text were split in two). Causal role (experimental) manipulation = perceived minor vs. major role of 1991 Kosovo referendum in subsequent Kosovo War; Self-reported Current Knowledge = current knowledge as measured in Studies 1-3 through self-report scales; Objective Current Knowledge = current knowledge as measured through true/false questions.

In summary, the expected effect of analogy exposure was not found, neither was the moderation by current knowledge. The manipulation in this study involved highlighting a past antecedent (the 1991 referendum for independence in Kosovo) and changing only the extent to which it played a single, major role (vs. a minor role) in the past outcome (the outbreak and escalation of violence). Perhaps the perception of causal antecedence, even minor, was enough to produce the effect, but the latter went undetected since the direction of causality was the same in both conditions (i.e., that the referendum contributed, more or less, to the outbreak of violence). Given that the other three studies all involved claims that differed in causal direction across conditions, this explanation is plausible. Thus this study is inconclusive, since we do not know whether the absence of significant differences relates to an absence of analogical effects on expectations about future outcomes, or to the presence of similar effects across conditions (which therefore remained undetected).

In summary, the four studies produced mixed results, with some finding only the interaction effect (studies 1 & 2), only the main effect (study 3) or neither (study 4). Yet, such mixed effects across a series of studies are bound to occur when a true effect is present (Francis, 2014)—especially when the Type I error rate is tightly controlled (e.g., via preregistration; Lakens & Etz, 2017) as was the case here. Thus, we conducted two separate “mini meta-analyses” (Goh, Hall & Rosenthal, 2016) in order to estimate more accurately the cumulative main and moderation effects across the four studies.

## Internal meta-analysis of the four studies

The meta-analyses were conducted on the effects obtained based on standardized data, using the R-based package metafor (Viechtbauer, 2017). We only assessed the meta-analytic effects on the main DV representing the difference in expectations about negative and positive future outcomes, as this measure was present in all studies. Given the high methodological similarities across studies, fixed-effects meta-analyses seemed adequate to account for sampling variation, but we also ran random-effects meta-analyses to see how the effect would generalize (Goh et al., 2016). The latter analysis was computed using the Sidik-Jonkman method, which is suitable for small samples (IntHout, Ioannidis & Borm, 2014). The results appear in ***Table 6***.

**Table 6:** Two internal meta-analyses of cumulative main and moderation effects of exposure to historical analogy on predictions across Studies 1–4.


	STUDIES				INTERNAL META-ANALYSES	
	
	1 (N = 132)	2 (N = 392)	3 (N = 361)	4 (N = 397)	FIXED-EFFECTS (N = 1282)	RANDOM-EFFECTS (N = 1282)

Main effect of analogy on predictions	–.13	.00	–.14**	.02	–.04 [–.09, .01]	–.05[–.14, .03]

Interaction effect Analogy × Current Knowledge on predictions	.17^†^	.12*	.09^†^	–.02	.07** [.02, .13]	.08^†^ [–.00, .16]

Simple effect at -1SD of current knowledge	–.32**	–.11	–.22***	.04	–.12***[–.17, –.07]	–.15^†^ [–.29, .01]

Simple effect at +1SD of current knowledge	.02	.14^†^	–.05	.00	.03 [–.02, .09]	.03^†^ [–.06, .12]


*Note*: All effect sizes are Pearson’s r. The random effect meta-analysis was obtained using the Sidik-Jonkman method. *** p < .001, ** p < .01, * p < .05, ^†^ p < .10. 95% confidence intervals are provided in brackets.

Regarding the main effect of analogies on predictions, the meta-analytic effect was very small and not significant regardless of the method used to assess it (r = –.04; Cohen’s d = –.08). The cumulative moderation effect by current knowledge was relatively bigger in size and significantly different from 0, or close to significance, depending on the method used (r = .07; d = .14). A meta-analysis of each simple effect at minus and plus 1 SD of current knowledge further indicated that the effect of analogy exposure on predictions was significantly different from zero only at lower levels of current knowledge (r = –.12; d = –.24), but not at higher ones (r = .03; d = .06). Since these findings were uncovered in the context of preregistered studies using sequential analyses that carefully controlled for the Type I error rate (except in the first study), the cumulative evidence appears reliable enough to be interpreted as supporting the presence of a small, but true, moderation effect by current knowledge on analogy-based predictions.

## General discussion

In this paper, we conducted four studies to examine whether exposure to a specific “lesson” involving causal claims between a past event and its outcome influences expectations about ongoing, real-life situations. The studies tested this hypothesis using different past and current real-life events. Studies 1 and 2 found that the expected analogical effect on predictions became stronger as current knowledge decreased. Given that those studies invoked different lessons derived from different events (the 1919 Versailles Treaty vs. the 1938 Munich Agreement), it was possible that the effects were related to other differences between the two events. Study 3 eliminated this possibility by using a text describing the same past events (the 1998 US military airstrikes against Al Qaeda and the subsequent 9/11 attack in 2001) and manipulating only the perceived causal relation between the two. Moreover, that study used a within-subject design by collecting measures before and after the manipulation to assess the change in predictions. Participants in that study expected a more negative outcome to the current intervention against ISIS after being told that past US airstrikes had been responsible for the 9/11 attack, compared to participants who were told those airstrikes had been effective in preventing an even worse attack. The moderation by current knowledge was only marginally significant in this case. Finally, study 4 tested whether presenting as minor (vs. major) the causal role of the focal antecedent in bringing about a past outcome would undermine the analogical effect on predictions. However, neither the main, nor moderation effects were found in this case. An internal meta-analysis across all four studies indicated that the effect of exposure to a past lesson on expectations became stronger as current knowledge decreased.

While small, the pattern of effects is consistent with previous studies on social judgment (Read, 1983; 1984) indicating that the presence of causally relevant features in both the source and target affects behavioral predictions for newly encountered target individuals. The present studies extend these findings by showing that such analogy-based predictive effects occur in the context of multifaceted and ambiguous real-life events—even without prompting participants into making the analogy. Rather, simply making available a specific “lesson” involving a particular causal claim about the past can change expectations regarding the future of the target situation—presumably because the perceived causal relation in the source had been analogically transferred to the target. Such spontaneous analogical effects are in line with previous studies demonstrating that the availability of a particular analogical source can influence memory representations of a target issue (Perrott et al., 2005), judgments and decisions in a hypothetical conflict (Gilovich, 1981), and support for a current political party (Smeekes et al., 2014).

In our case, the effect of the historical analogy was not universal as participants with higher current knowledge tended to rely less on the available analogy. While the literature on the topic does not report such a moderator, this discrepancy may be due the type of targets used. Other studies used hypothetical targets (Gilovich, 1981; Read, 1983; 1984), and well-known political parties (Smeekes et al., 2014). Variability in participants’ knowledge about the target issue was therefore completely controlled. In our case, the targets were real-life, ongoing situations with uncertain outcomes. Lack of current knowledge in this context may give rise to perceived uncertainty, which increased reliance on the analogy as a heuristic strategy (Tetlock, 1998; Vertzberger, 1986). This explanation is consistent with the positive correlation found in studies 2 and 3 between current knowledge and participants’ confidence in their predictions. Higher levels of current knowledge might also suggest familiarity with the target situation and the possibility to rely on that knowledge when making predictions. Read’s (1983) finding supports this interpretation: Individuals tend to rely on the available analogies only in the absence of a clear rule. We tried to disentangle some of these explanations in Study 4 by adding a measure of objective knowledge, but the results were inconclusive and merit further inspection to define this apparent boundary conditions of analogical effects.

Moreover, since awareness of the manipulation did not appear to play a role in these results (see *Appendix F*), the moderation by current knowledge is unlikely to simply reflect compliance from less knowledgeable participants with perceived experimental demands. Similarly, the analogical effect on predictions did not seem to depend on the perceived similarity between the past and present situations: While this ran contrary to our preregistered hypothesis in Study 3, this is in line with previous studies showing that effects of historical analogies on judgments (Gilovich, 1981) and memory representations (Perrott et al., 2005) occur even when participants do not explicitly perceive similarities between the source and target.

Finally, given that these findings were uncovered within highly educated samples, it is possible that this effect is actually stronger in the less-knowledgeable general population confronted with analogical messages from political figures—especially if those analogical messages involve particularly valued self- (or group) relevant past events. Conversely, our samples, while highly educated in general, are not necessarily experts on current affairs. Yet, the reliance on historical analogies for decision making has been well-documented among “elite” policymakers (e.g., Khong, 1992), who should be quite knowledgeable about current issues. Gilovich (1981) also showed that even students of political sciences were prone to be influenced by subtle historical analogies when making foreign-policy decisions. Thus, the fact that we only found analogical effects for less knowledgeable participants does not preclude the possibility that extremely knowledgeable people could also rely on historical analogies for predictions. In other words, it could be that both extremely low and extremely high levels of current knowledge result in stronger analogical effects.^2^ No evidence of such quadratic interaction effect by current knowledge was found in any of our four studies (see results highlighted in yellow in *Appendix G*)—perhaps because we lacked statistical power. Nonetheless, it would be worth investigating the conditions under which more (vs. less) knowledgeable people tend to rely on historical analogies for their predictions. Overall, the fact that the existing literature had not uncovered such a moderator by current knowledge further highlights the importance of studying the psychological effects of historical analogies in real-life, political situations where their use have been most documented and where they seem ecologically most prevalent, if we are to account fully for their specificities.

Notably, the effect was consistently found only on one measure, which constrained participants’ predictions for all possible outcomes to sum up to 100%. This instruction, common in the hindsight bias literature (Fischhoff, 1975), was used in order to force participants’ choices and avoid the situation where every outcome would be judged as equally likely—a legitimate concern given people’s general tendency to perceive the future as relatively open, uncertain, and positive (Weinstein, 1980). The constraint of this measure allowed us to assess a shift in participants’ willingness to consider the occurrence of a specific future negative outcome after exposure to the historical analogy. This shift would have otherwise gone unnoticed given the biases in future thinking. Therefore the experimental manipulation affected only this measure systematically; whereas the other measures were unable to reveal a similar shift in participants’ predictions. Future studies could use subtler, indirect measures (e.g., assessing how likely people are to invest money in their predictions) to assess changes in future expectations while incentivizing individuals to bypass typical biases in future thinking. Overall, the fact that exposure to historical analogies can shift, even minimally, the subjective likelihoods that people attach to real-life, future outcomes points to the potential political consequences of this phenomenon—especially given how prevalent such historical analogies are in public, political and media discourses.

## Implications and future research

By manipulating the perceived lessons of the past made available, the present research reproduces the ecology of political arguments based on historical analogies (Paris, 2002). In such arguments, the analogical source is often more detailed than the way in which it relates to the target (Blanchette & Dunbar, 2000); the listener is left with the task to draw the suggested analogical implications. In this sense, the experimental paradigms we used replicate those conditions: Only the past lesson was made available without specifying how it mapped to the target. This emphasizes the fact that similarity, here in terms of causal structure, is not a fixed feature of the events themselves. Rather it can be manipulated by making different causal claims about the source. This draws attention to the political arguments involved behind such historical analogies. In the end, both the moderation by current knowledge as well as the overall small and volatile nature of the effects found in the present research (as attested by the between-studies variation) suggest that the question of whether exposure to historical analogies affects predictions in political contexts is also ultimately a question of the conditions under which individuals will “buy” into the political claim that the past and present share causally structural elements that allow for meaningful lessons to be drawn.

## Footnotes

Without the covariate in the model, this main effect was smaller in size but still significant at the adjusted alpha level, F(1,359) = 5.84, p = .016, η^2^p = .016.

We wish to thank the editor, Dr. Alain Van Hiel, for suggesting this possibility to us.
